# Valproic Acid Regulates HR and Cell Cycle Through MUS81-pRPA2 Pathway in Response to Hydroxyurea

**DOI:** 10.3389/fonc.2021.681278

**Published:** 2021-08-27

**Authors:** Benyu Su, David Lim, Zhujun Tian, Guochao Liu, Chenxia Ding, Zuchao Cai, Chen Chen, Fengmei Zhang, Zhihui Feng

**Affiliations:** ^1^Department of Occupational and Environmental Health, School of Public Health, Cheeloo College of Medicine, Shandong University, Jinan, China; ^2^School of Health Sciences, Western Sydney University, Campbelltown, NSW, Australia; ^3^College of Medicine and Public Health, Flinders University, Bedford Park, SA, Australia; ^4^School of Public Health and Management, Wenzhou Medical University, Wenzhou, China

**Keywords:** breast cancer, VPA, HU, pRPA2, MUS81, cell cycle, HR

## Abstract

Breast cancer is the primary problem threatening women’s health. The combined application of valproic acid (VPA) and hydroxyurea (HU) has a synergistic effect on killing breast cancer cells, but the molecular mechanism remains elusive. Replication protein A2 phosphorylation (pRPA2), is essential for homologous recombination (HR) repair and cell cycle. Here we showed that in response to HU, the VPA significantly decreased the tumor cells survival, and promoted S-phase slippage, which was associated with the decrease of pCHK1 and WEE1/pCDK1-mediated checkpoint kinases phosphorylation pathway and inhibited pRPA2/Rad51-mediated HR repair pathway; the mutation of pRPA2 significantly diminished the above effect, indicating that VPA-caused HU sensitization was pRPA2 dependent. It was further found that VPA and HU combination treatment also resulted in the decrease of endonuclease MUS81. After MUS81 elimination, not only the level of pRPA2 was abolished in response to HU treatment, but also VPA-caused HU sensitization was significantly down-regulated through pRPA2-mediated checkpoint kinases phosphorylation and HR repair pathways. In addition, the VPA altered the tumor microenvironment and reduced tumor burden by recruiting macrophages to tumor sites; the Kaplan-Meier analysis showed that patients with high pRPA2 expression had significantly worse survival. Overall, our findings demonstrated that VPA influences HR repair and cell cycle through down-regulating MUS81-pRPA2 pathway in response to HU treatment.

## Introduction

Breast cancer is a common form of malignant tumor in women, about 2 million new cases are diagnosed each year (1). Chemotherapy is one of the mainstays of oncological treatment for breast cancer but is associated with adverse effects. To reduce the prevalence of adverse effects while enhancing the cytotoxic effect of chemotherapeutics on killing tumor cells, drug combination is commonly used (2).

In recent years, histone deacetylase inhibitors (HDACi) have been widely studied as a possible adjuvant or neoadjuvant to chemotherapy (3, 4). Specifically, valproic acid (VPA) - a class I and II HDACi used in the treatment of epilepsy and as a mood stabilizer for bipolar disorder (5–7) - has demonstrated the ability to inhibit the growth of breast cancer cells while also exhibiting a radio sensitizing effect at a safe therapeutic dose (6, 8–13). Hydroxyurea (HU), is a common chemotherapeutic for hematological malignancies such as polycythemia vera, melanoma, and head and neck cancer (14–17). The combination of VPA and HU has been demonstrated to have a synergistic effect in killing tumor cells (15–18), the molecular mechanism of action involves inhibition of Replication Protein A2 (RPA2) hyperphosphorylation-mediated DNA repair pathway (14).

RPA2 is the key subunit of the homologous recombination (HR) repair mechanism induced by DNA replication fork stagnation (19, 20). RPA2 comprises of multiple critical Ser/Thr residues that are phosphorylated sequentially in response to genotoxic stress (21). Ser33 phosphorylation is mediated by the ataxia telangiectasia and Rad3-related protein (ATR) (22, 23), which in turn promoted Thr21, Ser4 and Ser8 phosphorylation. Phosphorylation of Ser4/8 produces the most hyperphosphorylated form of RPA2 (24, 25). Studies have shown that phosphorylated RPA2 (pRPA2) is essential for HR repair as it is required for cell cycle checkpoints (26); directly interact with Rad51 (14, 27); and both RPA2 phosphorylation and HR repair occur mainly in the S- and G2-phases of the cell cycle (28, 29). Our previous studies have demonstrated that pRPA2 is specifically involved in the repair of DNA replication fork stagnation or collapse induced by HU (14, 30).

In this study, using different cell systems, an animal model of breast cancer, and human tissue samples, we systematically explored whether other mechanisms of action are involved in the observed cytotoxic effect of VPA and HU. We discovered that VPA influences HR repair and cell cycle through MUS81-pRPA2 pathway.

## Materials and Methods

### Cell Culture

Wild-type pRPA2 (wt-pRPA2) and hyperphosphorylation mutant RPA2-p (mu-pRPA2, S4A/S8A/S11A/S12A/S13A/T21A/S33A) cells were stably established as described in our previous publication (14). MCF10A cell line was transformed by the environment carcinogen DMBA (MCF10A-DMBA) as described elsewhere (31), MCF10A-DMBA cells were cultured with Dulbecco’s Modified Eagle’s Medium (DMEM)/Nutrient Mixture F-12 Ham (D9785, Sigma) combined with 5% horse serum (26050088, Gibco), 100ng/ml Cholera toxin (C8052, Sigma), 20ng/ml epidermal growth factor (E5036, Sigma), 0.5µg/ml hydrocortisone (614157, Sigma), 10µg/ml human insulin (I9278, Sigma), and 1% Penicillin-Streptomycin (V900929, Sigma). MCF-7, wt-pRPA2 and mu-pRPA2 cells were maintained in DMEM medium with 10% fetal bovine serum (FBS; Gibco).

### Drug Treatment of Cancer Cells

The VPA+HU treatment was previously described (14). In brief, the cells were pretreated with 0.5mM VPA (Sigma) for 24h or 48h before 2mM HU (Sigma) was added and cultured for another 18h, before subject to further experimental analysis.

### Clonogenic Survival Assay

The clonogenic survival assay method was described elsewhere (9, 32). The number of cell colonies (≥50 cells per clone) was counted and cell survival was presented as the cell survival fraction (SF), with SF = (the number of clones/seeded cells)/plating efficiency (PE).

### Comet Assay

Performed according to the manufacturer’s instructions (Trevigen Company), DNA damage was detected by alkaline and neutral comet assay.

### SiRNAs and Transfections

Knockdown of MUS81 in MCF-7 cells was performed by transfecting 200pmol siMUS81 (Genepharma) (5′-ACGCGCUUCGUAUUUCAGATT-3′ and 5′-UCUGAAAUACGAAGCGCGUTT-3′) (5′-GCAGGAGCCAUCAAGAAUATT-3′ and 5′-UAUUCUUGAUGGCUCCUGCTT-3′) or corresponding amounts of non-targeting control siRNA (Genepharma) with Lipofectamine 2000 (12566014, Thermo Fisher). After 24–48h, transfection mixture was removed and cells were stimulated with HU. Efficient knockdown was confirmed by western blotting.

### Cell Cycle Analysis

10μM of 5-Bromo-2-deoxyUridine (BrdU) (B5002, Sigma) was added to the cells 30min before the end of treatment, after which the cells were harvested and fixed in 70% ethanol overnight at -20°C. The cells were subsequently incubated with 0.4mg/ml of pepsin (Sigma) in 2M hydrochloride acid for 30min and neutralized with 0.1M sodium borate for 15min (Fisher Company). The cells were then washed and further incubated with the primary antibody of anti-BrdU (B44, BD). After washing, the cells were incubated with a secondary antibody of AlexaFluor 594-labeled goat anti-mouse IgG (Molecular probes). The nucleus was stained with 4′,6-diamidino-2-phenylindole (DAPI; Sigma) for cell cycle analysis by flow cytometry.

### Treatment of Animals and BrdU Incorporation

The female Sprague-Dawley (SD) rats used in this study, weighed between 200 and 250g, were obtained from Jinan Peng Yue Experimental Animal Breeding Co., Ltd (Jinan, CN). All animal experimental procedures were approved by the Shandong University Human and Animal Ethics Research Committee (81472800, approved March 2014). DMBA was dissolved in purified corn oil and adjusted to the concentration of 20mg/ml. Intragastric gavage (i.g.) was performed on SD rats with a single dose of 1ml DMBA-oil solution. During the experiment, the body weight was measured weekly. Meanwhile, breast palpation was performed on rats 3-4 times a week to check for tumor. About 40-60 days after gavage, primary tumors could be detected around the breast. The rats were randomly divided into four groups: untreated control, VPA, HU, and VPA+HU. The untreated group animals were treated with 0.9% saline. The rats in the VPA and VPA+HU groups received VPA [200mg/kg Intraperitoneal injection (IP), once a day] for 10 days. Four hours after the administration of VPA, HU (400mg/kg, IP, once a day) was administered to rats in the HU and VPA+HU groups for 10 days. The BrdU was injected IP to rats (100mg/kg) 24h before tissue harvest. On the 66th day after the end of the VPA and HU treatment, the rats were humanely euthanized pursuant to the animal ethics approval.

### Histopathological Analysis

The tumors were fixed in 4% paraformaldehyde solution for 48h, then embedded in paraffin and sectioned (5µm). Paraffin-embedded tumor tissue sections were deparaffinized in xylene and rehydrated in graded ethanol solution. Sections were counterstained with hematoxylin and eosin (HE) and observed under light microscope.

### Western Blot and Immunofluorescence Analysis

Western blot and immunofluorescence were performed as described previously (9, 32). The primary antibodies were anti-ATR (2790S, 1:800), anti-ATR (13934, 1:1000), anti-CHK1 (12908, 1:500), anti-phospho-CHK1 S317 (2344, 1:1000 for western blot; 1:150 for immunofluorescence), anti-WEE1 (sc-5285, 1:500), anti- pCDK1 (Y-15) (4539, 1:1000), anti-phospho-RPA2 Ser4/Ser8 (A300-245A, 1:2000 for western blot; 1:500 for immunofluorescence), anti-Rad51 (PC130, 1:1500), anti-Rad51 (sc-398587, 1:100), anti-γH2AX Ser139 (05-636, 1:1500), anti-53BP1 (NB100-304, 1:2500), anti-MUS81 (11018-1-AP, 1:1000), anti-Phospho-Histone H3 (Ser10) (66863-1-Ig, 1:2000), anti-phospho-RPA2 S33 (TA312067S, 1:1000), anti-CDK1 (CY5176, 1:1000) and anti-GAPDH (TA-08, 1:2000). Secondary antibodies included the goat anti-rabbit IgG horseradish peroxidase conjugated and goat anti-mouse IgG-horseradish peroxidase conjugated IgG (Pierce) in addition to the AlexaFluor 594-labeled goat anti-mouse IgG and AlexaFluor 488-labeled chicken anti-rabbit IgG (Molecular Probe). The images from the immunofluorescence assays were viewed at 100× magnification with a Zeiss Axio observer inverted fluorescence microscope (3858000984).

### Immunohistochemistry Analysis

Paraffin-embedded sections were deparaffinized and rehydrated as described above. Heat-induced epitope retrieval was performed. Endogenous peroxidase activity was inactivated by incubation in 3% hydrogen peroxide for 15min. Sections were incubated in goat serum for 1h. Following this, the slides were incubated in primary antibodies anti-F4/80 (123101, 1:200), anti-53BP1 (NB100-304, 1:1000), anti-phospho-CHK1 S317 (O14757, 1:100), anti-phospho-RPA2 Ser4/Ser8 (A300-245A, 1:1000), and anti-Rad51 (PC130, 1:500) overnight at 4°C, before incubation with biotinylated secondary antibody (1:300 dilution) for 1h then 30min in the ABC kit. The slides were incubated in diaminobenzidine (DAB), counterstained with hematoxylin, and sections were observed under light microscope.

### Statistical Analysis

Continuous values were expressed as mean ± standard deviation (SD). The unpaired two-tailed t-test was utilized to compare the groups. The Shapiro-Wilk test was used for the normality test. Correlation analysis of classification data was carried out by the chi-square test. All statistical analyses were performed using GraphPad Prism 6 (GraphPad Prism Software) or IBM SPSS Statistics for Windows, Version 25.0 software. Statistically significant differences were set at *P* < 0.05.

## Results

### Distribution of pRPA2 in Human Breast Cancer and Para-Carcinoma Tissues

**Figure 1:**
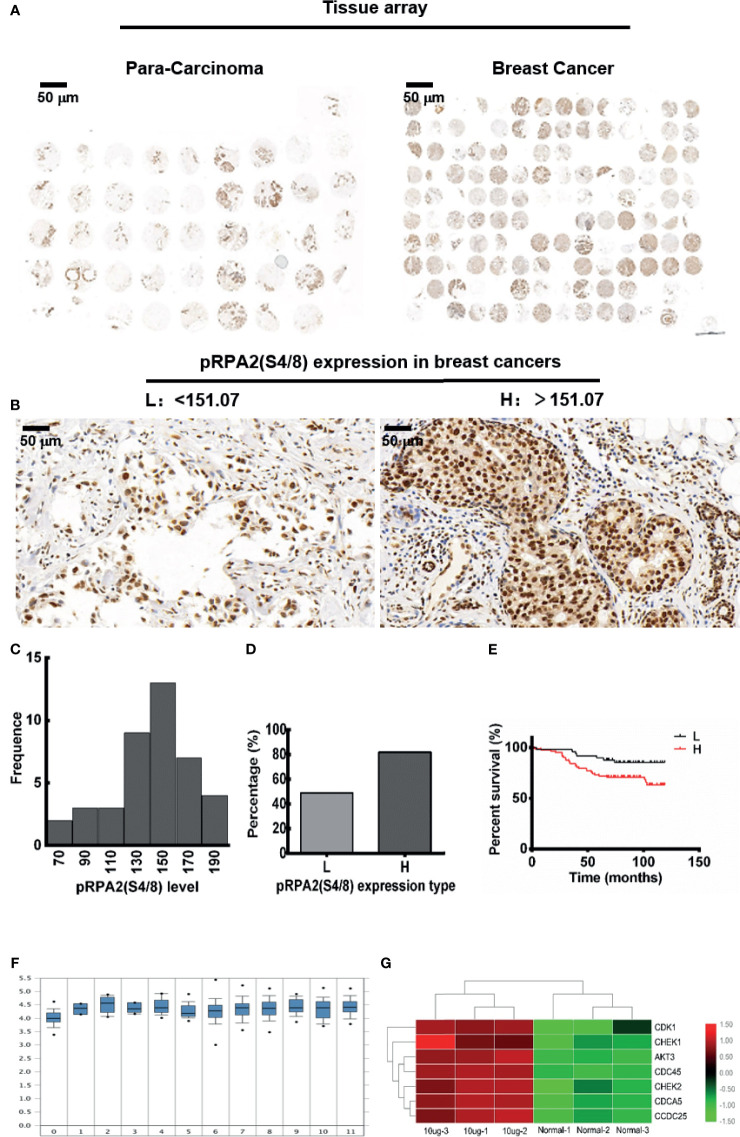
The distribution of pRPA2 in human tumor tissues and para-carcinoma tissues. **(A)** The tissue array of 45 para-cancerous tissue samples and 140 cancerous tissue samples. **(B, D)** The expression level of pRPA2 in tumor tissues was divided into two types: low-level type (L: pRPA2 < 151.07) and high-level type (H: pRPA2 > 151.07). **(C)** Shapiro-Wilk test (*P* > 0.05) suggests that the distribution of pRPA2 in paracancer tissues did conform to the normal distribution. **(E)** Kaplan-Meier analysis showed that patients with high pRPA2 expression had significantly worse survival. **(F)** Curtis dataset showed that the expression of the RPA2 gene in breast cancer tissues is higher than that in normal tissues. **(G)** The difference of cell cycle checkpoint kinase genes between DMBA-induced tumor cells and normal cells.

Our previous studies have proved that pRPA2 at S4/8 plays an important role in VPA sensitization chemotherapy (14), therefore, we want to explore the expression of pRPA2 at S4/8 in human tissues. We collected a total of 45 samples of para-cancerous tissues and 140 samples of cancerous tissues from Shanghai Outdo Biotech Company (Shanghai, China) in accordance with the ethics approval from Taizhou Hospital in Zhejiang province (**Figure 1A**). We first examined the levels of pRPA2 expression in the para-carcinoma tissues. The Shapiro-Wilk test (*P* > 0.05) indicated that the distribution of pRPA2 in the tissues did conform to the normal distribution (**Figure 1C**), therefore the 95% confidence interval was used. The normal medical reference range of pRPA2 in the paracancer tissues was 131.79 - 151.07. The expression levels of pRPA2 were divided into two types: low-level type (L: pRPA2 < 151.07), and high-level type (H: pRPA2 > 151.07) (**Figure 1B**). As shown in **Figure 1D**, the proportions of L and H types are 37.4% and 62.6% respectively, and pRPA2 is expressed predominately in the H type. The Curtis dataset in **Figure 1F** showed that the expression of the RPA2 gene in breast cancer tissues is higher than that in normal tissues. Kaplan-Meier analysis showed that patients with high pRPA2 expression had significantly worse survival (**Figure 1E**).

### VPA-Induced Breast Cancer Cell Death Is Dependent on pRPA2 in Response to HU Treatment

**Figure 2:**
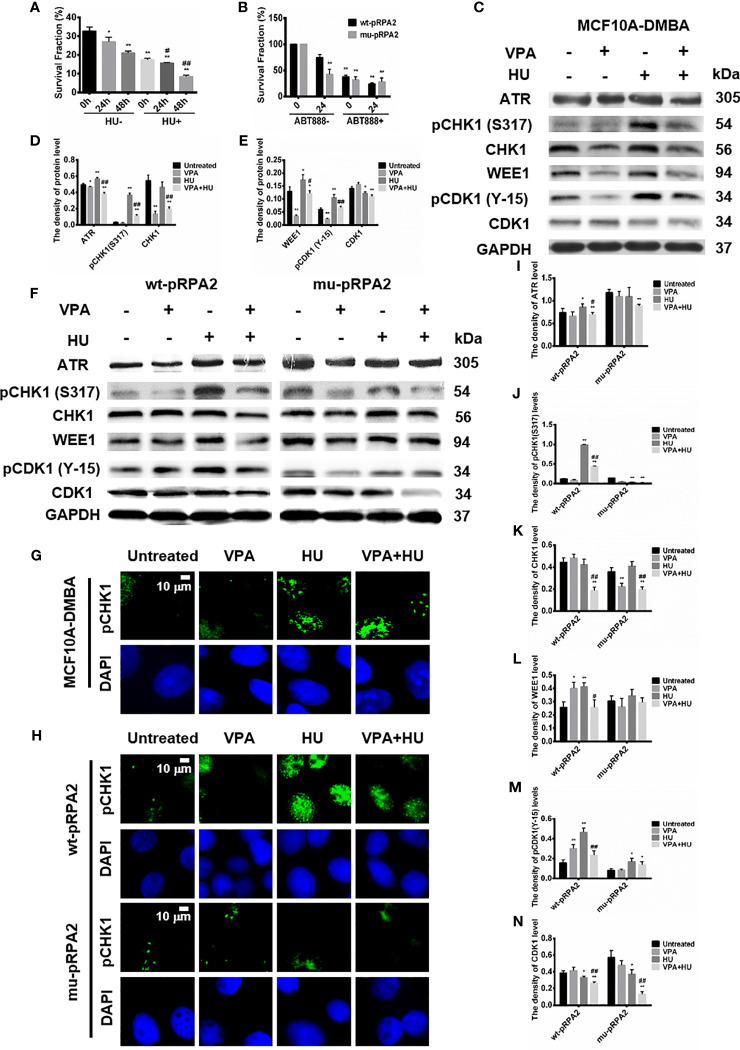
pRPA2 plays a key role in VPA-induced breast cancer cell death and VPA-inhibited checkpoint kinase phosphorylation. **(A, B)** Clonogenic survival in MCF10A-DMBA, wt-pRPA2 and mu-pRPA2 cells treated with VPA, HU, or combination of VPA and HU. **(C, F)** The levels of ATR, pCHK1 (S317), CHK1, WEE1, pCDK1 (Y-15) and CDK1 were detected by immunoblotting in MCF10A-DMBA, wt-pRPA2 and mu-pRPA2 cells treated as indicated in Fig. 1. **(D, E)**, **(I–N)** Quantification of the relative protein levels. GAPDH was used as loading control. **(G, H)** The pictures presented pCHK1 (S317) nuclear signal in MCF10A-DMBA, wt-pRPA2 and mu-pRPA2 cells. Each data point in the graph was from three independent experiments. Compared with the untreated group, *P < 0.05, **P < 0.01; compared with the HU group, ^#^ *P* < 0.05, ^##^ *P* < 0.01.

We previously demonstrated that intracellular replication breakage occurs with 2mM HU treatment for 18h (14, 30). As shown in **Figure 2A**, the survival fraction of MCF10A-DMBA cells treated by VPA+HU cells significantly decreased compared with the other groups (*P* < 0.01), indicating that VPA is capable of enhancing tumor cells sensitivity to HU.

VPA-induced cancer cell death may be associated with damage to pRPA2-mediated HR repair (14, 30). To test this hypothesis, we used the theory of synthetic lethality (SL) (33) to explore whether the HR mechanism of cells is affected. Due to the presence of cellular Poly (adenosine diphosphate (ADP)-ribose) polymerase inhibitors (PARPi), the cell cannot initiate the base excision repair (BER) pathway, so the two independent DNA damage repair pathways, HR and BER, are deficient and is fatal to the cancer cells (34). Hence, we employed ABT888, a typical PARPi, to test for MCF7 cells expressing mu-pRPA2 and wt-pRPA2 survival.

As shown in **Figure 2B**, the combined VPA and ABT888 inhibited wt-pRPA2 cell growth and killed tumor cells (*P* < 0.01). Before ABT888 and VPA treatment, the survival fraction in the untreated cells expressing mu-pRPA2 decreased by 49.22% (*P* < 0.01) as compared to the untreated cells expressing wt-pRPA2, indicating that the pRPA2 is critical to DNA repair. In contrast, there were no statistically significant differences in the cell survival rate between the ABT888 group and VPA+ABT888 group (*P* > 0.05), suggesting that pRPA2 is critical to replication forks repair, and without pRPA2, the effect of VPA is significantly reduced. We, therefore, concluded that VPA and ABT888 can synergistically kill tumor cells by inhibiting the HR and BER process, and the pRPA2 may be the potential target of VPA.

### VPA Can Inhibit pRPA2-Mediated Checkpoint Kinases Phosphorylation in Response to HU Treatment

Disturbances in the process of DNA replication forks and DNA damage activate checkpoint kinases, including ATR and checkpoint kinase-1 (CHK1) (35, 36). Obstructed replication forks activate kinases that promote cell cycle arrest and the intra S-phase checkpoint (35), which prevent fatal premature transitions of cells with incompletely replicated or damaged DNA into mitosis (37). WEE1 kinase is capable of phosphorylating cyclin-dependent kinase (CDK1) (38). ATR/pCHK1 and the WEE1/pCDK1 signal pathway regulates DNA replication origin firing during the S-phase and transition into the G2/M phase (4, 39, 40).

We next examined whether VPA influences checkpoint kinases phosphorylation. The MCF10A-DMBA cells were treated with HU, which impedes the progression of replication forks and activates checkpoint kinases. We found that the cell cycle checkpoint kinase CHK1 and CDK1 genes were increased in the cells (**Figure 1G**).

However, the combination of VPA and HU significantly diminished ATR, CHK1, WEE1, pCDK1 at Y-15, CDK1, pCHK1 at S317 and pCHK1(S317) nuclear signal in the cells (**Figures 2C–E** **, G**).

We further explored whether VPA can regulate checkpoint kinases phosphorylation in the wt-pRPA2 and mu-pRPA2 cells. The results showed that the combination of VPA and HU significantly decreased levels of ATR, pCHK1, CHK1, WEE1, pCDK1 and CDK1 in the wt-pRPA2 cells (**Figures 2F, I–N**). However, HU-alone could not increase the levels of ATR, pCHK1, WEE1, pCDK1 and CDK1 in the mu-pRPA2 cells, and there was no statistical difference between HU-alone and VPA+HU groups (**Figures 2F, I–N**). The results indicated that pRPA2 is capable of regulating checkpoint kinases phosphorylation. Furthermore, we also detected the pCHK1(S317) nuclear signal in wt-pRPA2 cells and mu-pRPA2 cells, and the results were consistent with the western blot results (**Figure 2H**). Hence, we concluded that VPA suppresses the cell cycle checkpoint kinases phosphorylation in response to replicative stress in several cellular systems.

### S Phase Slippage to G2/M Promoted by VPA Is Related to pRPA2 After HU Treatment

**Figure 3:**
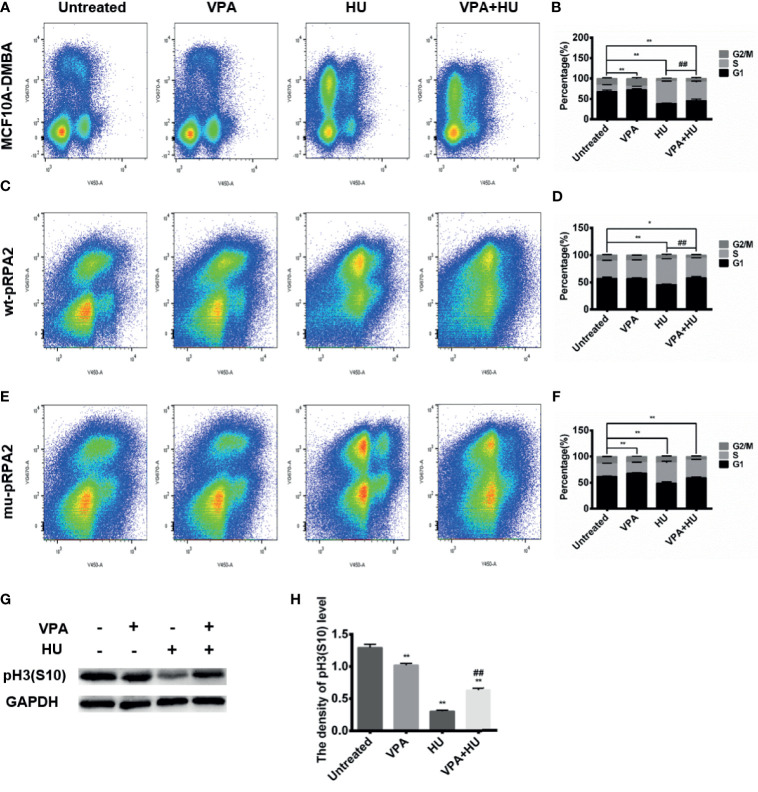
VPA promotes S phase slippage. **(A–F)**:MCF10A-DMBA, wt-pRPA2 and mu-pRPA2 cells were treated with VPA and/or HU. Cell cycle analysis is shown as mean ± SD (n = 3). Statistical significance is displayed for cells in the S-phase. **(G)** The level of pHistone H3 (S10) was detected by immunoblotting. **(H)** Quantification of the relative protein levels. GAPDH was used as loading control. Each data point in the graph was from three independent experiments. Compared with the untreated group, *P < 0.05, **P < 0.01; compared with the HU group, ^##^ *P* < 0.01.

Cell cycle progression is restricted due to activated checkpoint kinases. We hypothesized that VPA may potentially disturb this mechanism. As shown in **Figures 3A, B**, HU arrested MCF10A-DMBA cells in the S-phase and delayed their transition into the G2/M-phase, VPA induced a cell cycle arrest in the G1-phase. As expected from its ability to block checkpoint kinase phosphorylation (**Figure 2F**), VPA reduced HU-treated MCF10A-DMBA cells in the S-phase and led the cells to slide into the G2-phase (**Figures 3A, G, H**). Following VPA and HU treatment, breast cancer cells had increased levels of Histone H3 (S10), indicating an increase of mitotic cells, compared with HU alone (**Figures 3G, H**). Hence, we deduced that breast cancer cells exposed to VPA+HU escape from the HU-induced S-phase block and traverse into G2-phase and catastrophic injury.

**Figure 4:**
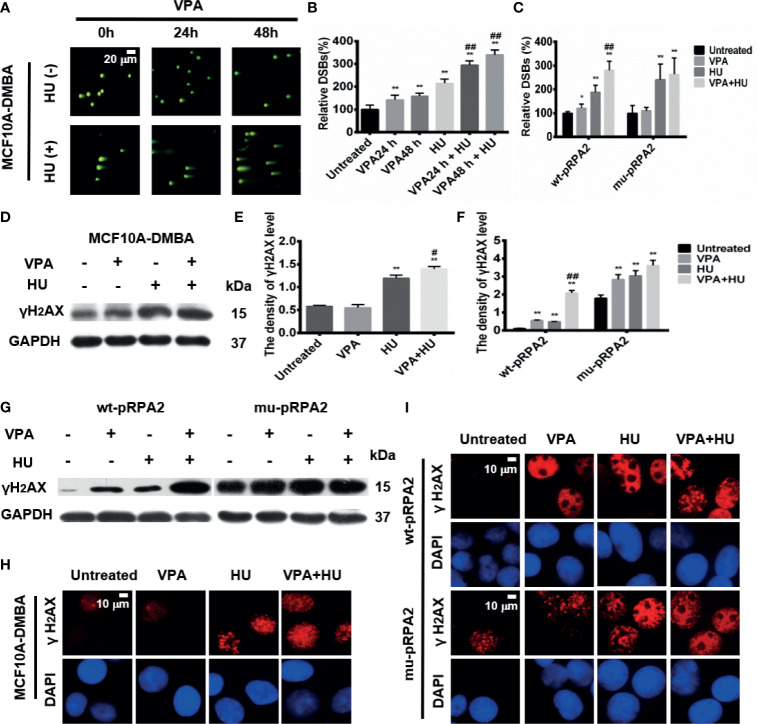
VPA influences DNA DSBs after HU treatment in breast cancer cells. **(A)** The MCF10A-DMBA cells treated as indicated in Fig. 1 with comet tail presented in the pictures using neutral come assay. **(B, C)** Relative DSBs of cells were analyzed. **(D, G)** The level of γH2AX was detected by immunoblotting in MCF10A-DMBA, wt-pRPA2 and mu-pRPA2 cells. **(E, F)** Quantification of the relative protein levels. GAPDH was used as loading control. **(H, I)** γH2AX foci formation in MCF10A-DMBA, wt-pRPA2 and mu-pRPA2 cells. Each data point in the graph was from three independent experiments. Compared with the untreated group, *P < 0.05, **P < 0.01; compared with the HU group, ^#^ *P* < 0.05, ^##^ *P* < 0.01.

When DNA is replicated in the S-phase and during chromosome segregation in the M-phase, cells are particularly vulnerable to genome instability and DNA damage. The pCHK1 and pCDK1 activation defect, and persistent γH2AX seen in mu-pRPA2 cells suggest that DNA damage accumulates when these cells decrease in S-phase stasis in response to replication stress (**Figures 4G, I**). To determine whether RPA2 phosphorylation is also important to prevent damaged cells from entering mitosis, we next analyzed wt-pRPA2 and mu-pRPA2 cell cycle profiles. As shown in **Figures 3C–F**, VPA reduced HU-treated wt-pRPA2 cells in the S-phase, but the S-phase of mu-pRPA2 cells treated with HU was reduced by 20.71% as compared with the corresponding group in the wt-pRPA2 cells. There was no statistical difference between the HU and VPA+HU group in the mu-pRPA2 cells. These data indicated that RPA2 phosphorylation is essential for maintaining S-phase checkpoint arrest, and pRPA2 may be the potential target of VPA.

### VPA Enhances HU-Induced DNA DSBs Through the Inhibition of pRPA2 Level in Tumor Cells

Since HU treatment leads to collapsed replication forks to one-ended DNA double-strand breaks (DSBs), we next investigated VPA’s effect on DSBs under HU treatment using the comet assay. The neutral comet assay results showed that the comet tail length in the MCF10A-DMBA cells treated with VPA+HU significantly increased compared with other groups (**Figures 4A, B**, *P* < 0.01). These results further indicated that the combination of VPA and HU enhances MCF10A-DMBA cells’ sensitivity. The results were verified using the alkaline comet assay (**Supplementary Figures 1A, B**, *P* < 0.01).

The findings presented in **Supplementary Figure 1E** and **Figure 4C** demonstrated that VPA and/or HU significantly increased the comet tails in wt-pRPA2 cells (*P* < 0.01), while the comet tails in the HU-alone group of mu-pRPA2-cells were significantly longer than the corresponding group of wt-pRPA2-cells. No significant difference in the tail length between the HU and VPA+HU groups in the mu-pRPA2 cells was observed. The findings suggest that pRPA2 is important to replication forks repair, and without pRPA2, the effect of VPA is significantly reduced. Similar results were obtained using the alkaline comet assay (**Supplementary Figures 1C, D**, *P* < 0.01).

The DSBs marker, γH2AX was used to detect DNA damage in MCF10A-DMBA cells. The results showed that the protein level of γH2AX in the VPA+HU group was significantly higher than each of the single drug treatment groups in the cells (**Figures 4D, E**). Immunofluorescence assay showed that VPA enhanced the HU-induced positive signal of the cells with nucleus γH2AX foci in the cells (**Figure 4H**).

We also checked for γH2AX in the wt-pRPA2 and mu-pRPA2 cells. Western blot results showed that VPA and/or HU significantly increased the level of γH2AX in wt-pRPA2 cells, while the level of γH2AX in the HU-alone group of mu-pRPA2 cells was significantly higher than the corresponding group of wt-pRPA2 cells (*P* < 0.01). However, in the mu-pRPA2 cells, there was no significant difference in the level of γH2AX between the HU and VPA+HU groups (**Figures 4G, F**). The level of γH2AX was detected by an immunofluorescence assay, and the results were consistent with the western blot results (**Figure 4I**).

In summary, we demonstrated using different cell lines that VPA enhances HU-induced DNA DSB breaks, and pRPA2 plays a crucial role in DNA repair.

### VPA-Induced HU Sensitization Is Associated With the Decrease of pRPA2-Mediated HR Repair Function

To further elucidate the molecular mechanisms of how VPA can sensitize tumor cells to HU treatment, we hypothesized that VPA disrupts the HR repair signaling pathway.

**Figure 5:**
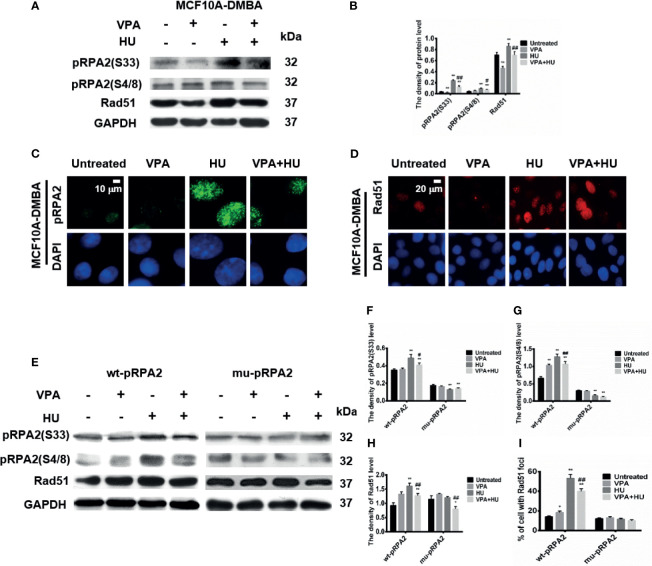
VPA influences the pRPA2 and Rad51 in cell system. **(A, E)** The levels of pRPA2(S33), pRPA2(S4/8) and Rad51 were detected by immunoblotting in MCF10A-DMBA, wt-pRPA2 and mu-pRPA2 cells treated as indicated in Fig. 1. **(B, F–H)** Quantification of the relative protein levels. GAPDH was used as loading control. **(C, D)** pRPA2(S4/8) and Rad51 foci formation in MCF10A-DMBA cells. **(I)** Quantification of the Rad51 foci. Each data point in the graph was from three independent experiments. Compared with the untreated group, *P < 0.05, **P < 0.01; compared with the HU group, ^#^ *P* < 0.05, ^##^ *P* < 0.01.

The results in **Figures 5A–D** showed that pRPA2 at both sites of S33 and S4/8 and Rad51 levels in the VPA+HU group were significantly decreased compared to HU only treatment in the MCF10A-DMBA cells. The results suggested that sensitization is caused by VPA interfering with the pRPA2 at both sites of S33 and S4/8, mediated by Rad51-dependent HR pathway.

To further verify the above results, we also assessed pRPA2 at both sites of S33 and S4/8 and Rad51 in the wt-pRPA2 and mu-pRPA2 cells. The results showed that VPA+HU significantly decreased pRPA2 and Rad51 induced by HU in the wt-pRPA2 cells (**Figures 5E–H**). However, HU did not increase the pRPA2 and Rad51 in the mu-pRPA2 cells, and there was no statistically significant difference between the HU and VPA+HU groups. We also detected the positive signal of the cells with pRPA2 and Rad51 foci in wt-pRPA2 cells or mu-pRPA2 cells. Consistent findings were obtained *via* western blot (**Figure 5I** and **Supplementary Figures 2A, B**). These results indicated that VPA induced cell sensitivity by interfering with the pRPA2 mediated Rad51-dependent HR pathway, and pRPA2 is critical to this pathway.

### VPA-Caused HU Sensitization Is Associated With Endonuclease MUS81

Methyl methane sulfonate ultraviolet sensitive gene clone 81 (MUS81) plays an important role in maintaining genome stability and replication fork integrity (41).

Notably, recent studies have found that the expression level of MUS81 is closely related to the evolution of various cancers (42, 43). The crossover junction endonuclease MUS81 interacts with EME1 and EME2 to form a DNA structure-specific endonuclease with substrate preference for branched DNA structures with a 5’-end at the branch nick (44). In addition, MUS81 protein abundance increases in cells following exposure to agents that block DNA replication (45, 46).

We next examined how VPA reduces pRPA2 in response to HU treatment. We hypothesized that VPA interferes with endonuclease MUS81-mediated nuclease activation, and result in the replication forks failure to trigger the functional signal pathway after HU treatment.

**Figure 6:**
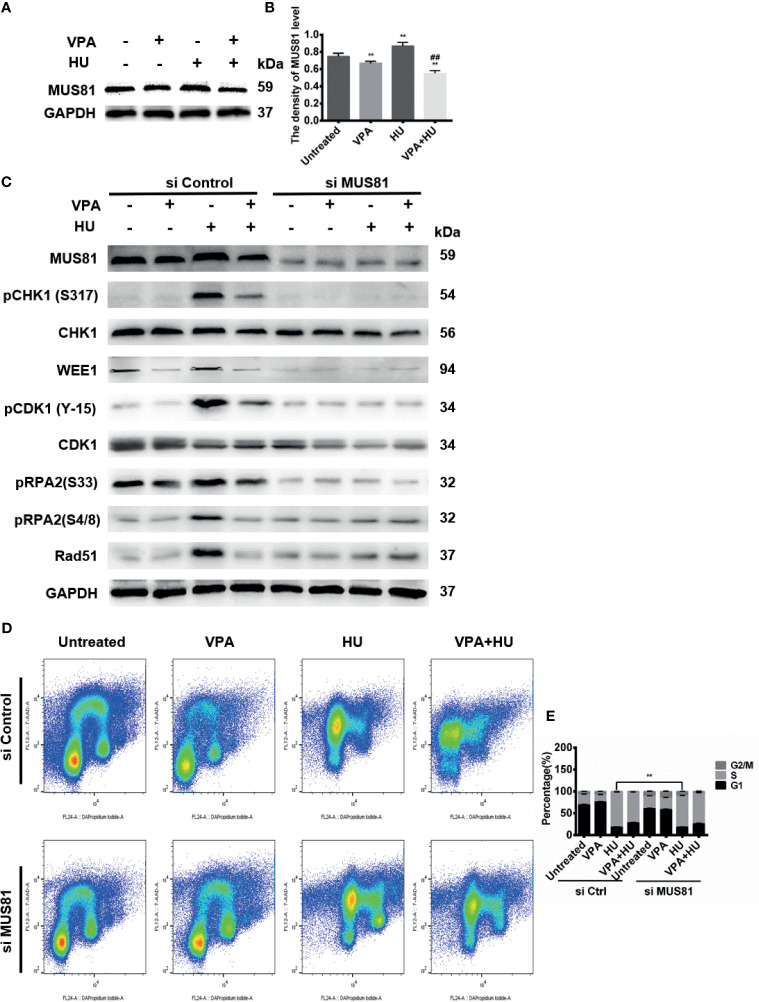
VPA-caused HU sensitization is associated with endonuclease MUS81. **(A)** The level of MUS81 was detected by immunoblotting in MCF-7 cells treated as indicated in Fig. 1. **(B)** Quantification of the MUS81 protein levels. GAPDH was used as loading control. **(C)** MCF-7 cells were transiently transfected with indicated siMUS81. After 24 h, cells were treated with 2 mM HU and 0.5 mM VPA. Protein detection was performed by immunoblot. **(D, E)** MCF-7 cells were treated with 2 mM HU, 0.5 mM VPA or/and siMUS81. Cell cycle analysis of the DNA content of MCF-7 cells using flow cytometry. Cell cycle analysis shown as mean ± SD (n = 3). Compared with the untreated group, **P < 0.01; compared with the HU group, ^##^ *P* < 0.01.

As shown in **Figures 6A, B**, the combination of VPA and HU significantly diminished MUS81 in MCF7 cells (**Figures 6A, B**). After knocking down MUS81 by its siRNA, pRPA2 at both sites of S33 and S4/8 was down-regulated (**Figure 6C**), indicating that the level of pRPA2 was regulated by MUS81.

To next investigate the role of MUS81 in HR repair and cell cycle, we decreased MUS81 with its siRNA in MCF-7 cells, and treated the cells with VPA and HU, then analyzed the checkpoint kinases phosphorylation, cell cycle progression and HR repair proteins. As demonstrated in **Figure 6C**, after down-regulation of MUS81, pCHK1, WEE1, pCDK1, CDK1, pRPA2 and Rad51, VPA appeared to lose its effect in HU sensitization. The cell population at S-phase in siMUS81 cells treated with HU was reduced by 10.88% as compared with the corresponding group in the si control cells, a significant difference in the S-phase was not detected between HU and VPA+HU groups, consistent with the results of checkpoint kinases in siMUS81 cells (**Figures 6D, E**). The data indicated VPA can target MUS81-pRPA2-mediated checkpoint kinases phosphorylation and HR pathways for causing replication forks to fail to signal.

### VPA Sensitizes Tumor Tissues to HU Treatment in Rats *In Vivo*

**Figure 7:**
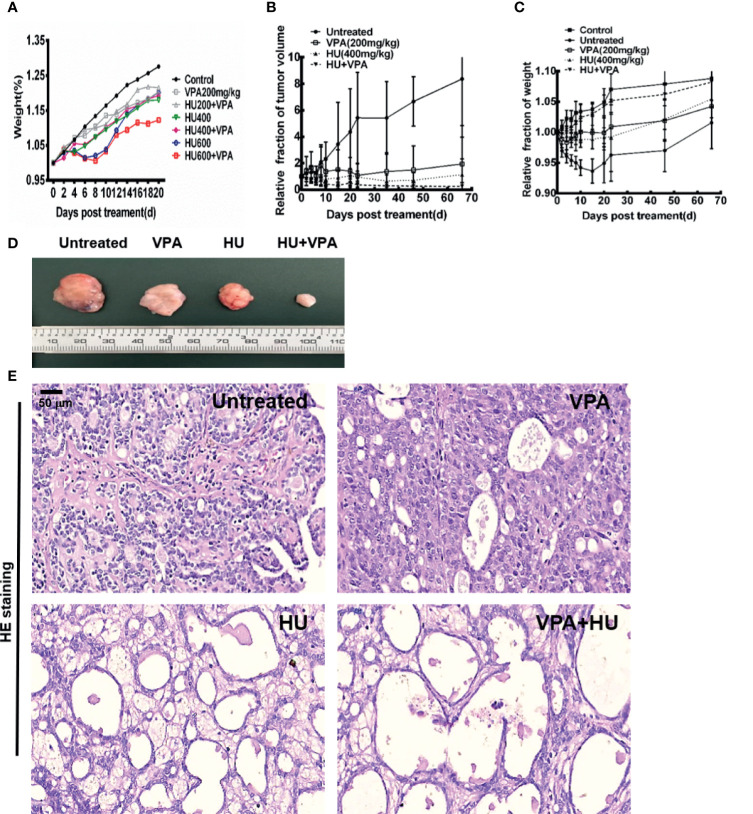
VPA sensitizes tumor to HU treatment in rats. **(A)** The effect of different doses of VPA and HU on the bodyweight of normal rats.**(B)** The changes of rat tumor volume in untreated control, 200mg/kg VPA, 400mg/kg HU, and VPA+HU groups. **(C)** The changes of rat body weight in different groups. **(D)** Photographs of tumor volume in untreated, VPA, HU, and VPA+HU groups. **(E)** The morphological change of tumor in groups after treatment.

For the next set of experiments, we used a primary rat model of breast cancer induced by DMBA, described in our previous studies (14, 47). It was reported in the literature that 150 to 300mg/kg of VPA was usually used for animal study (34, 48). In this study, 200mg/kg was employed. Previous study used 50mg/kg/day to 1500mg/kg/day over 10 days to study the toxic effect of HU on rats (49). In this study, three concentrations of 200, 400 and 600mg/kg HU were selected. As shown in **Figure 7A** and **Supplementary Tables 1, 2**, we found that under the condition of 400mg/kg HU not only effectively kills the tumor but also minimizes the side effects. Therefore, this dose was chosen for subsequent experiments.

We concurred with previous studies that VPA treatment produced a significant reduction in tumor volume (**Figure 7D**). This trend was more pronounced in the HU and VPA+HU groups (**Figure 7B**, *P* < 0.01). We observed that the untreated rats exhibited a large number of mammary duct dysplasia and interstitial fibrosis in the tumor tissue, and the acinar structure disappeared, presenting a typical tumor cell morphology (**Figure 7E**). Some vacuoles structures of tumor tissue were found in the VPA-alone rats; more vacuoles structures and necrotic cells occurred in the HU-alone rats. These changes were more obvious in the VPA+HU rats. There was no significant difference in the body weight between the untreated control and VPA+HU groups at the endpoint, indicating that VPA minimized the side effects of HU (**Figure 7C**). The above results indicated that VPA efficiently sensitizes primary tumor to HU treatment.

### VPA Influences Checkpoint Kinases, HR Repair, and Macrophages in Tumor Tissues

**Figure 8:**
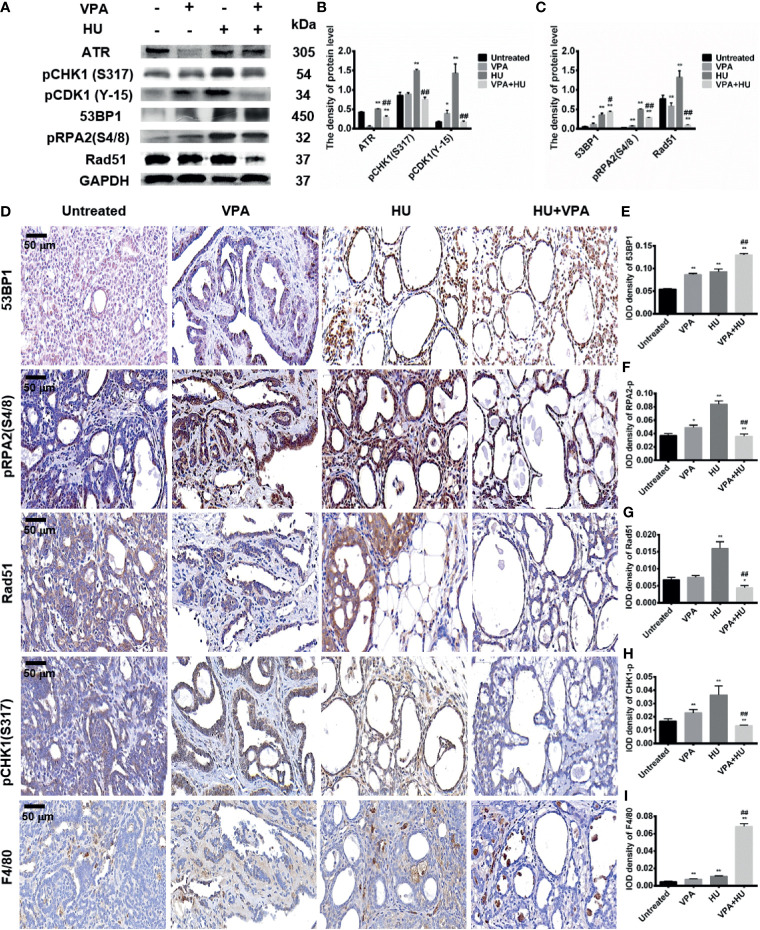
VPA influences checkpoint kinases phosphorylation and HR repair in tumor tissues. **(A)** The protein levels of ATR, pCHK1 (S317), pCDK1 (Y-15), 53BP1, pRPA2(S4/8) and Rad51 were detected by immunoblotting in tumor tissues. **(B, C)** Quantification of the relative protein levels. GAPDH was used as loading control. **(D–I)** The protein expression of 53BP1, pRPA2(S4/8), Rad51 and pCHK1 (S317) by immunohistochemistry staining in tumor tissues; Macrophages were detected in the tumor by the specific marker F4/80. IOD density of indicated proteins in immunohistochemistry photos was quantified by Image pro plus software. Each data point in the graph was from three independent experiments. Compared with the untreated group, *P < 0.05, **P < 0.01; compared with the HU group, ^#^ *P* < 0.05, ^##^ *P* < 0.01.

Based on the results of checkpoint kinase in the different cell systems mentioned above, we next verify the above results in rats *in vivo*. As demonstrated in **Figures 8A, B**, HU significantly increased, while VPA+HU significantly decreased, the protein levels of pCHK1 at S317 and pCDK1 at Y-15 in the tumor tissues. Furthermore, we also tested the expression of pCHK1 using immunohistochemistry analysis, and the results were consistent with the western blot results (**Figures 8D, H**).

The DSBs marker 53BP1 was used to detect DNA damage in tumor tissues. The results showed that the protein level of 53BP1 significantly increased in the HU group and VPA+HU group (**Figures 8A, C**). Similar results were obtained using immunohistochemistry analysis. More importantly, 53BP1 expression was only localized in the tumor cells of hyperplastic glands accompanying more vacuole structures and necrotic cells (**Figures 8D, E**). We further tested the pRPA2 at S4/8 and Rad51 protein levels, the key factor for HR. The pRPA2 and Rad51 were significantly decreased in the VPA+HU group (**Figures 8A, C**). Similar results were obtained using immunohistochemistry analysis (**Figures 8D, F, G**), indicating that HU blocks the replication fork of DNA and activates the repair function of HR, and pRPA2 and Rad51 in HR repair are inhibited by VPA+HU.

Recently, it was reported that TMP195, a class IIa HDACi, altered the tumor microenvironment and reduced tumor burden and pulmonary metastases by recruiting macrophages to tumor sites (50), we hypothesized that VPA might regulate macrophages recruitment in breast cancer metastasis. The F4/80, a specific macrophage marker, was used to detect the number and distribution of macrophages in the tumor tissues. We found that macrophages increased slightly in the VPA (*P* < 0.05) and HU (*P* < 0.01) groups. The VPA+HU group had a higher proportion of F4/80, and the macrophages were mainly distributed in the stroma and inside of the tumor gland (**Figures 8D, I**). Therefore, we concluded that VPA sensitizes tumor to HU treatment by stimulating the proliferation of macrophages and recruiting it into tumor tissues.

## Discussion

Chemotherapeutic drugs are widely used in the clinical treatment of breast cancer, but due to its toxic adverse effects, finding an effective chemotherapeutic sensitizer is garnering clinical interest (51, 52). We and others have demonstrated that VPA can sensitize breast cancer cells, pancreatic cancer cells, and melanoma cells to HU (14, 18, 53). While mechanism of action had been proposed, the precise pathway is yet to be thoroughly investigated.

In this study, we first established a homologous pair cell-line expressing wt-pRPA2 or mu-pRPA2, MCF10A-DMBA cells, and animal models that simulated the development of human primary tumors. Through the animal and *in vitro* cell culture, we demonstrated the combined treatment of VPA and HU was effective in inhibiting tumor growth, and the use of VPA alone could also inhibit tumor growth, indicating that pretreatment with VPA can enhance the response of breast cancer to HU. These results confirmed previous studies using breast cancer cell lines (14).

It was reported that VPA sensitized tumor cells to chemotherapy through apoptosis and autophagy (8, 54). Our studies found that disruption to the cell cycle and DNA repair functions are also important mechanisms for VPA sensitization; specifically, VPA decreases checkpoint kinase phosphorylation, promotes S-phase slippage, and inhibits pRPA2-mediated HR repair pathway following HU treatment.

Our results from the wt-pRPA2 and mu-pRPA2 cells confirmed that pRPA2 plays a vital role in activating the checkpoint kinases, regulating cell cycle progression, and specifically participating in the HR repair. In the present study, we found that the mutation of pRPA2 (S4A/S8A/S11A/S12A/S13A/T21A/S33A) in MCF-7 cells significantly decreased MCF-7 cell survival, and ATR, pCHK1 (S317), CHK1, WEE1, CDK1, and pCDK1 (Y-15) could not be activated by HU, thus confirming that pRPA2 is required for checkpoint kinases and cell cycle profile. To the best of our knowledge, this is the first report to identify that WEE1/pCDK1 (Y-15) are regulated by pRPA2. In addition, VPA could not decrease the pCHK1 (S317) and pCDK1 (Y-15) checkpoint kinase phosphorylation in mu-pRPA2 cells. Therefore, we demonstrated that pRPA2 plays a pivotal role in the sensitization of VPA.

**Figure 9:**
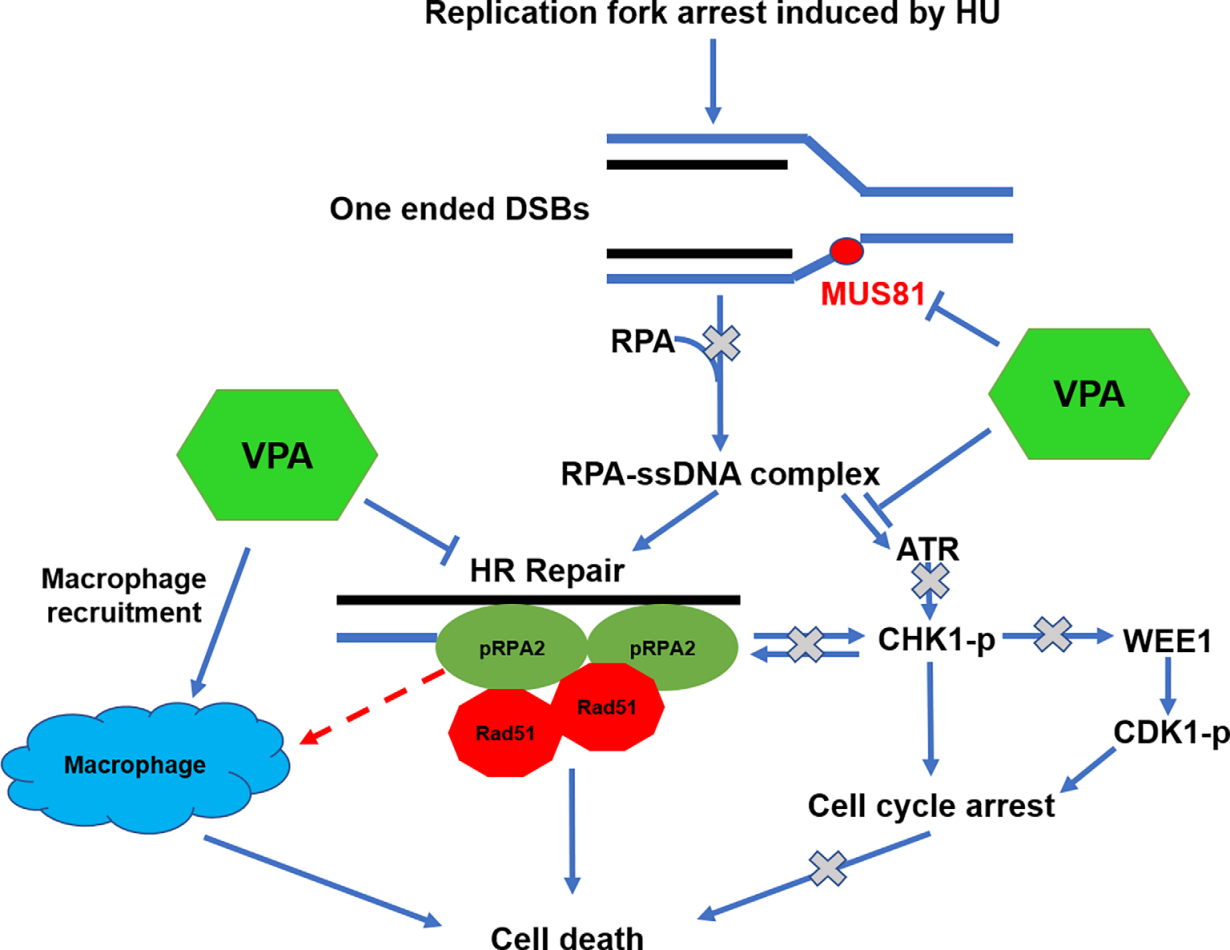
The hypothetical modulation model of VPA on tumor cells.

We specifically test for the action of VPA on S33- and S4/S8-RPA2 phosphorylation since the stagnant replication forks need to be modified by endonuclease for signaling to initiate DNA repair. MUS81 interacts with EME1 and EME2 to form a DNA structure-specific endonuclease and is involved in DNA repair, gene replication and cell growth (55). Our results corroborate that VPA interferes with the activation of MUS81 nuclease and prevents the signal transduction of the replication fork, thereby reducing the levels of pRPA2 at both S33 and S4/8 (**Figure 9**). To the best of our knowledge, this is the first report that the HDACi can interfere with MUS81.

We further documented the expression of S4/S8-RPA2 phosphorylation in human breast tumor and paracancer tissues by immunohistochemistry. We determined the medical reference range of pRPA2(S4/8), found the clinical association of pRPA2(S4/8) in human breast tumors, and identified the relationship between the expression level of pRPA2(S4/8) and patients’ survival. Specifically, patients with breast cancer tumors expressing high pRPA2(S4/8) had much worse survival. These results indicate that pRPA2(S4/8) represents a new potential predictive biomarker to identify patients who may respond to VPA and HU combined therapy.

Using the animal model, we found that macrophage-mediated immune response was also involved in the VPA-mediated therapeutic effects. Notably, in this study, compared with the HU-alone treatment, the combination of VPA and HU can stimulate macrophages and recruit the macrophages into the tumor site to inhibit tumor growth. These results suggested that VPA influences macrophages in the tumor microenvironment: it can effectively enhance the killing effect of chemotherapy on tumor tissue. Whilst the molecular mechanism involved in the regulation of macrophages is still unclear, our results confirm that pRPA2 regulates cell cycle checkpoint kinases and cell cycle, whether pRPA2 can affect the function of macrophages and the relationship between HR repair and tumor immune response will need to be further investigated.

In summary, the present study demonstrated that VPA influences HR repair and cell cycle through MUS81-pRPA2 pathway in response to HU and macrophages are involved in the regulation of VPA.

## Data Availability Statement

The original contributions presented in the study are included in the article/**Supplementary Material**. Further inquiries can be directed to the corresponding author.

## Ethics Statement

The studies involving human participants were reviewed and approved by The ethics committee of Taizhou Hospital in Zhejiang province. The patients/participants provided their written informed consent to participate in this study. The animal study was reviewed and approved by Shandong University Human and Animal Ethics Research Committee. Written informed consent was obtained from the individual(s) for the publication of any potentially identifiable images or data included in this article.

## Author Contributions

ZF: Conceptualization, validation, resources, writing - review and editing, supervision, project administration, and funding acquisition. BS: Methodology, software, formal analysis, investigation, writing - original draft, and visualization. ZT, GL, and CD: Validation and formal analysis. DL, ZC, CC, and FZ: Writing - review and editing. All authors contributed to the article and approved the submitted version.

## Funding

This study was supported by grants from the National Natural Science Foundation of China (No. 81472800), and the Key Technology Research and Development Program of Shandong (2019GSF108083).

## Conflict of Interest

The authors declare that the research was conducted in the absence of any commercial or financial relationships that could be construed as a potential conflict of interest.

## Publisher’s Note

All claims expressed in this article are solely those of the authors and do not necessarily represent those of their affiliated organizations, or those of the publisher, the editors and the reviewers. Any product that may be evaluated in this article, or claim that may be made by its manufacturer, is not guaranteed or endorsed by the publisher.
