# Supplementary figures and images for: Valproic Acid Regulates HR and Cell Cycle Through MUS81-pRPA2 Pathway in Response to Hydroxyurea
Source: Front Oncol. 2021 Aug 27;11:681278. doi: 10.3389/fonc.2021.681278 (PMC8429838; doi:10.3389/fonc.2021.681278)

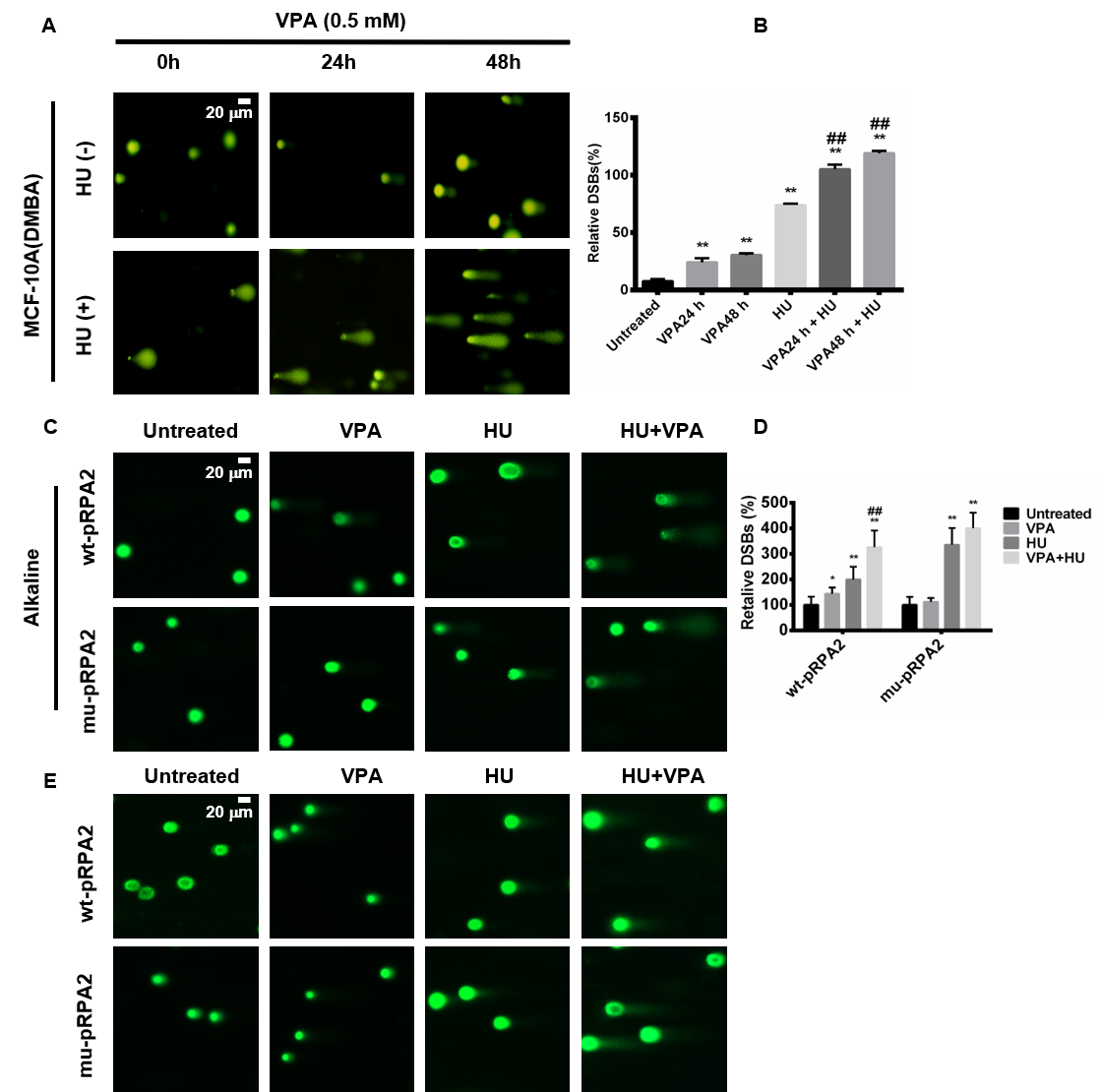

Supplement: Supplementary file 1 [file Image_1.tif]

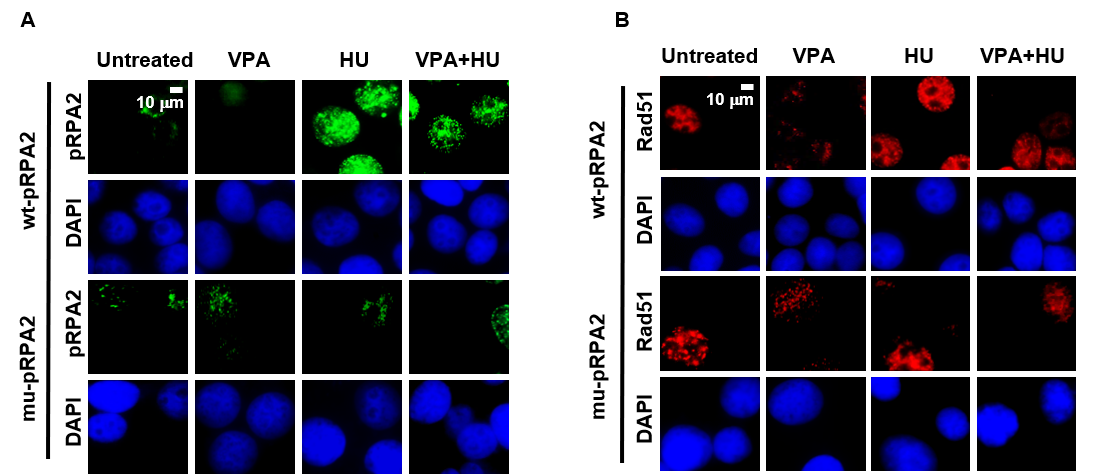

Supplement: Supplementary file 2 [file Image_2.tif]

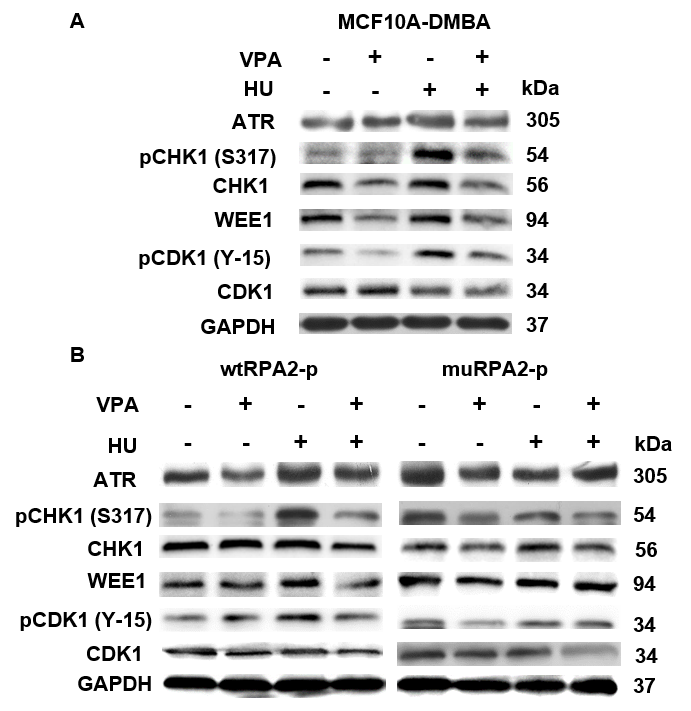

Supplement: Supplementary file 3 [file Image_3.tif]
