# Supplementary material for: Valproic Acid Regulates HR and Cell Cycle Through MUS81-pRPA2 Pathway in Response to Hydroxyurea
Source: Front Oncol. 2021 Aug 27;11:681278. doi: 10.3389/fonc.2021.681278 (PMC8429838; doi:10.3389/fonc.2021.681278)

**Table 1. Toxicity and mortality of rats at different doses**


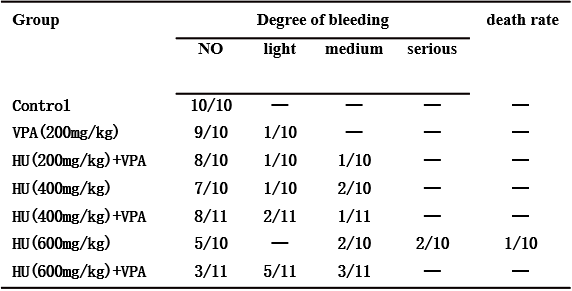


**Table 2. Toxicity and mortality of rats after 10 days of treatment**


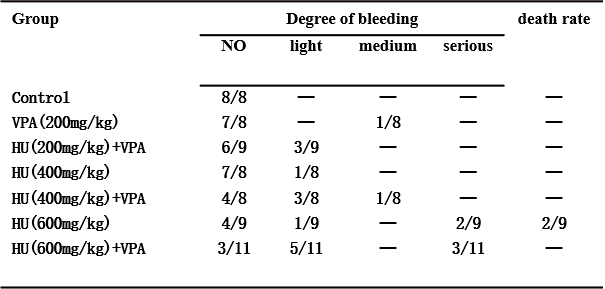

Supplement: Supplementary file 4 [file Table_1.docx]
